# Antioxidant defence barrier of great tit *Parus major* nestlings in response to trace elements

**DOI:** 10.1007/s11356-020-08495-9

**Published:** 2020-04-02

**Authors:** Beata Koim-Puchowska, Joanna M. Drozdz-Afelt, Robert Lamparski, Aleksandra Menka, Piotr Kaminski

**Affiliations:** 1 Department of Biotechnology, Kazimierz Wielki University, K.J. Poniatowski St12, 85-671, Bydgoszcz, Poland; 2 Department of Biology and Plant Protection, UTP University of Science and Technology, Prof. S. Kaliski St. 7, 85-796 Bydgoszcz, Poland; 3 Collegium Medicum in Bydgoszcz, Department of Ecology and Environmental Protection, Nicolaus Copernicus University, M. Skłodowska-Curie St. 9, 85-094 Bydgoszcz, Poland; 4 Faculty of Biological Sciences, Institute of Biotechnology and Environmental Protection, Department of Biotechnology, University of Zielona Góra, Prof. Szafran St. 1, 65-516 Zielona Góra, Poland

**Keywords:** Anthropogenic pollution, Oxidative stress, Antioxidant defence barrier, Trace elements, Nestlings

## Abstract

Metals can have direct and indirect effects on the generation of reactive oxygen species in wild birds. The aim of this work has been to examine the effect of exposure to trace metals (copper Cu, iron Fe, cobalt Co, manganese Mn) on oxidative stress biomarkers such as lipoperoxidation TBARS and level of superoxide dismutase SOD, catalase CAT, and reduced glutathione GSH in the livers and kidneys of great tit *Parus major* nestlings (*n* = 165, 63 broods) living in polluted environments associated with soda plants and agricultural activities (Kujawy region) and from a reference site (Tuchola Forest), both in the north of Poland. As we predicted, the level of TBARS in both organs of chicks from polluted areas was higher than in those from reference site. This could be connected with Fe concentrations, particularly in areas adjacent to soda plants (livers *R*_s_ = 0.49, *p* < 0.002; kidneys *R*_s_ = 0.69, *p* < 0.001). We also showed differences in the level of antioxidants depending on the environment. CAT activity was higher in nestlings from Kujawy than in those from Tuchola. Meanwhile SOD activity (both organs) and GSH levels (kidneys) were lower in the polluted area compared to the reference site. Concentrations of Cu, Fe, Co, and Mn may play a role in regulating the antioxidant system components’ activity.

## Introduction

Chemical elements are natural and biochemically active elements of the environment (Kabata-Pendias 2010; Tchounwou et al. 2012). However, the anthropogenisation of the environment, such as by increased industrial activity and agriculture practices, has significantly increased the pools of elements in ecosystems, resulting in disorders such as increased mortality of chicks and adult birds, decrease in breeding success, genetic changes, paler plumage, lower carotenoids, depletion of available food, and also alterations to habitat, as well as changes to the community structure or the ecological relationships between species (Dauwe et al. 2005; Eeva et al. 2006; Geens et al. 2009; Berglund et al. 2010; Pamplona and Costantini 2011; Eeva et al. 2012; Rainio et al. 2013; Pacyna et al. 2018). Small passerines, the great tit included, are considered ideal candidates for biomonitoring of the point environment, as it is a widespread species with a rapid metabolic rate and a small area of feed search, as opposed to nocturnal birds or fish-eating birds (Deng et al. 2007; Berglund et al. 2011). Nestlings are a good source of information about the state of the environment, because during their stay in the nest they do not change their location, and food is provided to them from the local environment by their parents (Peakall and Burger 2003). The research material to determine the degree of contamination primarily consists of parenchymatous organs (liver, kidney), feathers, excrement, and blood (Isaksson et al. 2005; Deng et al. 2007; Isaksson et al.; 2009; Martinez-Haro et al. 2011; Sánches-Virosta et al. 2015; Rubio et al. 2016; Turzańska-Pietras et al. 2018). The accumulation of metals in the organs of *P. major* depends on several factors, i.e.: their concentration in food (which mainly consists of insects), water, and air; the period of exposure; their interaction with other elements; their form; but also the rate of the bird’s metabolism and detoxification (Deng et al. 2007; Koivula and Eeva 2010). Many works have investigated *P. major* as a bioindicator of environmental pollution or measured the impact of heavy metals on different parameters determining the condition and reproductive success of birds (Dauwe et al. 2004; Dauwe et al. 2006). In recent years, a few papers have focused on oxidative stress as a secondary consequence of environmental pollution, including the effect of heavy metals (Koivula et al. 2011; Espín et al. 2014; Herrera-Dueñas et al. 2017; Stauffer et al. 2017; Sanches-Virosta et al. 2019, 2020). It is confirmed that chemical elements are involved in generating reactive oxygen species (ROS) such as hydroxyl radical (OH^-•^), superoxide radical (O_2_^-•^), or hydrogen peroxide (H_2_O_2_) (Koivula and Eeva 2010; Espín et al. 2014; de la Casa-Resino et al. 2015). Transition metals, e.g. Fe or Cu, may exist in more than one state of oxidation; hence, unpaired valence electrons allow them to participate in single-electron redox reactions. In biological systems, Fe(II) in particular catalyses the Fenton reaction, which creates hydroxyl radicals from hydrogen peroxide, while Fe (III) is regenerated via the Haber–Weiss reaction. A product of both of these reactions—hydroxyl radical (OH^-•^)—is known for having very strong oxidative properties with such biomolecules as DNA, lipids, and proteins (Costantini 2008). The disturbance of the balance between the amount of generated ROS and the efficient activity of antioxidant mechanisms is associated with the state of oxidative stress, which results in such dysfunctions as disintegrations of the permeability of membranes, modifications in heme synthesis and the content of haemoglobin, haemolysis, damage to nuclear and mitochondrial DNA, mutagenesis, carcinogenesis, or intensification of apoptosis (Ercal et al. 2001; Isaksson et al. 2009; Halliwell and Gutteridge 2015; Isaksson 2015). The concentrations of thiobarbituric acid reacting substances (TBARS) are used as biomarkers of lipoperoxidation (Koivula and Eeva 2010; Espín et al. 2017; Isaksson et al. 2017). This complicated process disturbs the functioning of the membrane: it decreases fluidity and increases leakage, damages membrane proteins, and inactivates receptors, enzymes, and ion channels (Halliwell and Gutteridge 2015). Birds are able to modify defence mechanisms in response to the degree of environmental pollution (Koivula and Eeva 2010; Isaksson 2015). In addition to the detoxification of elements, antioxidant mechanisms are activated (Isaksson et al. 2005). Antioxidative enzymes are the first line of the body’s defence against reactive oxygen species (Isaksson et al. 2005; Koivula and Eeva 2010; Ighodaro and Akinloye 2018). Catalase (CAT) and superoxide dismutase (SOD) are metalloproteins, and their function is associated with, respectively, H_2_O_2_ and O_2_^-•^ detoxification (Gurer and Ercal 2000; Glorieux and Calderon 2018). SOD occurs in three forms in eukaryotic cells: containing Cu and Zn in the cytoplasm, containing Mn in the mitochondrial matrix, and extracellularly with Cu and Zn (Koivula and Eeva 2010). In turn, CAT that contains Fe in hem is located to cellular peroxisomes, with particularly high concentration in the liver (Imrich et al. 2007). In addition to enzymes, small-molecule antioxidants such as reduced glutathione (GSH), composed of amino acid residues of glutamic acid, cysteine, and glycine, play an important role in the deactivation of ROS by binding with them. Additionally, GSH takes part in regeneration of other antioxidants, i.e. vitamin E, carotenoids, and also constitutes a reservoir of cysteine (Isaksson et al. 2005). The thiol group of reduced glutathione (GSH) is also a binding site for many metals, including iron, which blocks Fenton reactions and the formation of a hydroxyl radical (Koivula and Eeva 2010). Glutathione exists in two forms: reduced (GSH) and oxidised (glutathione disulfide GSSG), but the GSSG:GSH concentration ratio is also used as a biomarker of oxidative stress (Isaksson et al. 2005; Isaksson 2015).

In our work, we assumed that changes in concentrations of trace metals (Fe, Cu, Mn, Co) in disturbed ecosystems—especially due to the proximity of soda and landfills generated by a sodium factory, as well as intensive agricultural activity—could have an impact on ROS generation, and indirectly or directly stimulated or reduced the activity of antioxidant defence. We hypothesised that nestlings of *Parus major* located in polluted environments would exhibit oxidative damage and somewhat enhanced levels of antioxidant in comparison with unpolluted sites as results of a higher oxidative challenge. Birds activate many different antioxidants to protect themselves against oxidative stress (Berglund et al. 2007), and thus, we investigated the activity of: superoxide dismutase SOD, catalase CAT, concentration of reduced glutathione GSH, and also the content of thiobarbituric acid reactive substances TBARS to confirm the occurrence of oxidative stress in the livers and kidneys of great tit nestlings being bred in different types of environments. Simultaneously, we examined the concentrations of trace elements (Fe, Cu, Mn, Co), which not only induce oxidative stress but are also essential elements for correct development of nestlings and are a component of antioxidant enzymes. We examined the level of these elements in tissues of chicks which grow and feed in various types of pollution. These studies are also planned in order to understand the relationships between different antioxidant biomarkers and transition metals in the livers and kidneys of great tit chicks in polluted areas, as compared with control groups from Tuchola Forest.

### Study area

Studies were conducted in the area of the Inowrocław Ecological Hazards Region (Kujawy, Central Poland) and Tuchola Forest (Northern Poland). Two types of environment were studied in the county of Inowrocław in the Kujawy region (52°–53° N, 18°–20° E): (1) a strong degree of human impact, associated with sodium-industry activities and waste dumps (a), and (2) agriculture areas (b). The control environment (c) was chosen in an unpolluted area of the Tuchola Forest natural forest complex (53°40′–54° N, 17°30′–18°35′ E), characterised by a lack of highly developed industry, large afforestation, which is 48.8% (average for the province: 22.73%), and numerous lakes and rivers. We sampled nestlings at two sites in each environment. Sites 1 (52°46′47.2”N 18°06′24.7″E) and 2 (52°46′01.7”N 18°06′29.0″E) (environments A) located in the immediate vicinity of the soda plant in Janikowo and at the landfill site of the soda plant near Giebnia. Meanwhile, sites 3 and 4 (environment B) were located about 5 km from the soda plants. Nesting buildings were located near fields in the vicinity of the Notecki Canal (52°46′36.9”N 18°08′34.0″E) and Pakoskie Lake (52°47′35.1”N 18°05′08.5″E). The reference sites were located about 100 km north of the polluted areas (53°32′04.1”N 18°08′30.4″E and 53°32′10.6”N 18°07′48.6″E).

The study area in Kujawy was located on the border of two macrostructures of mesozoic tectonics: the Kujawski Wall and the Mogileńska Valley. The Kuyavian-Pomeranian underground mountain range is located under a blanket of quaternary and tertiary Cenozoic. This elevation is accompanied by Zechstein rock salt sediments, which in the Kujawy section of the embankment have been lifted in the form of salt columns. The salt pans are accompanied by salty sources and salty underground waters under hydrostatic pressure called “salines” (Piernik 2003). The salt deposits in this area provided the foundations for the development of the soda industry in Inowrocław and Janikowo. The products of these factories are soda ash, baking soda, evaporated salt, calcium chloride, salt chloride mixtures, and salt itself. The production of soda ash by the Solvay method produces, besides the intended products, huge amounts of waste—lime sludge with a lot of sodium and chlorine. Thus, the high saturation of soil sorbing complex by Na ions (Piernik 2012; Kamiński et al. 2016) and the salinisation of surface water and groundwater (Hulisz et al. 2017) in the Kujawy region are linked to the natural salts deposit, but especially to industrial wastes, e.g. calcium and iron compounds, silicates, aluminosilicates, and solutions of KCl, NaCl, NH_4_OH, Na_2_SO_4_, NaOH, MgCl_2_, and CaCl_2_ known as “sludge liquor”, stored in leaking earth tanks (settling ponds), which infiltrate into the substrate (Piernik 2003; Kamiński et al. 2016). Furthermore, environmental problems also include failures in pipelines draining the wastewater from the factory to the Noteć River and the Vistula River and delivering brine for soda production from the mine in Góra to the factory in Mątwy (Piernik 2003; Hulisz et al. 2017). Concentrations of Ca, Na, and Cl ions in surface waters, especially those contaminated with soda wastes and industrial brine, were many times higher than the highest permissible values of pollution for wastewater being conducted into waters and soil (Cl 1 g/dm, Na^+^ 0.08 g/dm) (Hulisz et al. 2017). Our previous studies confirm a high level of salinity (Ec > 14 mS), alkalisations (pH > 8) of soil, and destabilisation of Ca, Mg, Na, and Fe management connected with sodium factories and agriculture practices (Kamiński et al. 2012; Kamiński et al. 2016). It has been shown that alkaline soils are high in sorbent soils and contain large amounts of iron minerals, especially in hydrated amorphous form. They accumulate significant amounts of certain elements, making them inaccessible to plants. Any change in the chemical balance caused by, for example, rainfall acid precipitation or a decrease in the level of organic matter may cause the mobility and phytoavailability of the associated elements in the soil sorbing complex (Kabata-Pendia 2010). In particular, high concentrations (ppm) of chemical elements Na (821.81 ± 823.663; 451.04 ± 412.291), Ca (83,778.73 ± 104,267.017; 40,552 ± 38,357.177), and Fe (5724.14 ± 1541.227; 9312.59 ± 2272.352) in soil from areas located in the vicinity of sodium factories (Kamiński et al. 2012) create the risk of incorporating toxic elements into the trophic chain. The research of Kamiński et al. (2012) indicated the accumulation of Na, Ca, or Fe at the levels > 2000 mg/kg, > 13,000 mg/kg, and > 500 mg/kg, respectively, in plant organs from these contaminated areas. In addition, it has been concluded that Na, Ca, Cu, and also Fe may both stimulate lipid peroxidation and modulate activity of antioxidant enzymes: superoxide dismutase (SOD), catalase (CAT), and ascorbate peroxidase (APOX) of glycophytes in the Kujawy region. Subsequent ecosystem research in this area proved bioaccumulation of trace elements, in particular Zn, Cu, Mn, Co, and Cd, within the trophic chain: water–soil–plants–invertebrates (including insects, which constitute the main food for the chicks of *P. major*) (Kamiński et al. 2016). Also, studies conducted on human blood collected from people living permanently in this area indicate an increased level of toxic metals: Pb (0.0236 mg/L) and Cd (0.0008 mg/L) in comparison with the inhabitants of Tuchola (Pb, 0.014 mg/L; Cd, 0.0005 mg/L), although the concentration of Fe was lower in Kujawy (0.442 g/L) than in Tuchola (0.496 g/L). In addition, a relationship has been demonstrated between Cd, Pb concentration, and the activity of the oxidative stress parameters studied in the blood serum, including the activity of superoxide dismutase (SOD) (Wieloch et al. 2012).

Additionally, the area located in the vicinity of Inowrocław is dominated by very fertile black lands (II and III bonitation class) (Wieloch et al. 2012). Simultaneously, fertilisers used in crop cultivation and technological progress in agriculture are also a source of heavy metals (Kabata-Pendias 2010; Nagajyoti et al. 2010). The content of organic matter ranges from 8.76 to 16.40% (Kamiński et al. 2016), which stimulated the development of agriculture in this area.

To sum up, despite the new environmental technologies being introduced in both factories and agricultural activities, the past effects of the sodium salt industry are still visible in the alkalisation and salinity of soil, and ground and surface waters, but also in the bioaccumulation of chemical elements in the trophic chain (Kamiński et al. 2016; Hulisz et al. 2017).

## Material and methods

Investigations were carried out in two breeding seasons (2011 and 2012) from mid-April to July. In total, 145 nesting boxes were hung to implement the project (50 around the Janikosoda Plant in Janikowo and Giebnia; 50 around Pakości and the Notecki Canal; 45 in Tuchola Forest). We monitored the sites regularly, every other day, in order to obtain research material. The livers and kidneys were collected from nestlings (*n* = 165) in different growth phases (1st age group, 1–7 days; 2nd age group, 8–14 days; 3rd age group, 15–21 days) (Table 1). Such a partition was performed because nestlings show three successive stages of development during their stay in the nest: (1) intensive development of internal organs, (2) a sharp increase in biomass, and (3) a fall in growth rate, and even a fall in body mass that conforms with the regularities for this group of birds (Kaufman 1962; Keskpaik and Davydov 1967). Chicks were randomly collected from nest boxes inhabited by *Parus major*. The number of chicks collected from a single brood did not exceed three, according to the permit obtained from the General Nature Conservation Dept. (DONOOŚogiz-4200/III-13/44/08/aj). Nestlings were euthanised with isoflurane. The livers and kidneys were immediately dissected and placed on dry ice in Dewar flasks and transported to a laboratory. The collected material was stored at ˗80 °C until further analyses. In total, we collected data (livers and kidneys; *n* = 165) from 63 breeding nests (SM-18, AE-20, BT-25). Due to the small amount of material obtained from the youngest chicks, each research sample in this age group was a combination of organs of three chicks from the same brood. Thus our research considered 123 samples (Table 1).

**Table 1:** Number of samples analysed

Age of nestlings	Sodium manufacture (A)	Agricultures (B)	Control (C)	number of samples
1 (1–7 days)	6	8	7	21*
2 (8–14 days)	11	14	18	43
3 (15–21 days)	21	22	16	59
Total	38	44	41	123

*the sample determine organs received from three individuals from one hatch

### Concentration of chemical elements

The analysis of the concentration of chemical elements was preceded by homogenisation and mineralisation of samples. The Liver and kidney wet weight did not exceed 0.23 g and 0.1 g, respectively. Each piece of material (about 0.06 g of kidney and 0.1 g of liver) was dried at 50 °C to a constant mass and then homogenised in a porcelain mortar. The remaining material was used for biochemical analyses. The dried, powdered samples were placed in Eppendorf tubes and sent to an accredited laboratory—SGS Polska Sp. z o.o. Environment, Health and Safety in Pszczyna—for metal analysis. Mineralisation was done using the Berghof speedwave MWS-2 system (microwave pressure digestion unit with built-in in situ temperature measurement) to receive a clear solution. The contents of elements, ppm of dry weight (DW), were then determined using inductively coupled plasma mass spectrometry (ICP-MS AGILENT 7500 CE). The results were given in mg/kg DW (Wieloch et al. 2012). All determinations were made in the presence of 45Sc, 89Y, and 159 Tb as an internal standard to maintain apparatus stability and minimise matrix effects. Standard reference materials were not available for examined elements, and in-house controls and calibration curves were applied (Godwin et al. 2016).

### Superoxide dismutase activity SOD

The biological material was rinsed with phosphate buffered saline PBS. The tissues were homogenised in a solution of 20 mm of the buffer of HEPES (pH 7.2) containing 1 mm EGTA, 210 mm mannitol, and 70 mm of the sucrose per gram of tissue. The obtained homogenate was placed in test tubes and centrifuged at 1500 × g for 5 min at 4 °C. The supernatant was kept in the freezer at − 80 °C. The activity of superoxide dismutase was measured by the standard in a homogenate of the tissue using SOD Assay Kit (Cayman Chemical Co., No. 706002). All of the procedures were adopted in accordance with the methodology specified by Liu (1996) and by Maier and Chan (2002). This method utilises a tetrazolium salt for detection of superoxide radicals generated by xanthine oxidase and hypoxanthine. One unit of SOD is defined as the amount of enzyme needed to exhibit 50% of dismutation of superoxide radical. The absorbance was read at 450 nm using a plate reader. The results were interpreted comparatively with the standard well-known concentration. The results were given in U/ml.

### Catalase CAT activity and reduced glutathione GSH concentration

Biological material was rinsed with phosphate buffered saline PBS. Tissues were homogenised in a solution of 50 mm of potassium phosphate (pH 7.0) containing 1 mm EDTA per gram of tissue. The obtained homogenate was placed in test tubes and centrifuged at 10,000  ×  g for 15 min at 4 °C. The supernatant intended for the assay of CAT activity was kept in the freezer at − 80 °C. The equal volume of MPA (metaphosphoric acid, Sigma-Aldrich) was added to samples intended for the assay of reduced glutathione and then mixed. Successively, samples were subjected to incubation at room temperature for 5 min and then were centrifuged at 2000  ×  g for 2 min, and supernatant was collected and then kept at − 20 °C. The samples before the assay of CAT activity were diluted with the buffer and joined in the kit (15 × kidney; 25 × liver).

The catalase activity was measured in serum using CAT Assay Kit (Cayman Chemical Co., No. 707002). All of the procedures were adopted in accordance with the methodology specified by Johansson and Borg (1988) and by Wheeler et al. (1990). This method uses colorimetric measurement of formaldehyde, produced in the reaction of CAT with methanol in the presence of H_2_O_2_; 4-amino-3-hydrazino-5-mercapto-1,2,4-triazol (chromogen). One unit of CAT is defined as the amount of enzyme that will cause the formation of 1.0 nmol of formaldehyde per minute at 25 °C. The absorbance was read at 540 nm using a plate reader. The results were interpreted comparatively with the standard well-known concentration and were showed in nmol/min/ml.

The GSH concentration was marked using a standardised kit (Cayman Chemical Co., No. 703002). In this method, the glutathione reductase is used for the quantification of GSH. The SH group of GSH reacts with DTNB (5,5′-dithio-*bis*-2-(nitrobenzoic acid) producing yellow TNB (5-thio-2-nitrobenzoic acid). The mixed disulfide, GSTNB (between GSH and TNB), that is concomitantly produced is reduced by glutathione reductase to recycle GSH and produce more TNB. The rate of TNB production is directly proportional to the GSH concentration in the sample. The measurement of the absorbance of TNB at 405 nm provides an accurate estimation of GSH in the sample. Before the realisation of the assay on every ml of sample, 50 μl of the TEAM (trietanoloamina, Sigma-Aldrich Just. T58300) was added for the purpose of increasing the pH of the samples and as a result creating a suitable environment for further reactions. Glutathione concentration in the investigated samples was presented in μm.

### Lipid peroxidation

Lipid peroxidation was approximated using a thiobarbituric acid reactive substances assay according to Hermes-Lima et al. (1995). This method is based on the reaction of a degradation product of lipid peroxidation with thiobarbituric acid (TBA) at high temperature and acidity to generate a coloured adduct that is measured spectrofluorometrically.

Dry liver and kidney were homogenised in 1.1% phosphoric acid and reacted with TBA solution (7% phosphoric acid and 0.1 mm butylatedhydroxytoluene BHT). For blanks, tissues were homogenised as described above, with the exception of the use of 3 mm HCl instead of TBA. The samples were heated to 100 °C for 15 min before the addition of butanol. Furthermore, the samples were mixed for 3 min and centrifuged at 2000  ×  g for 15 min. Absorbance in the organic phase was measured at 532 and 600 nm. The samples were compared to the blanks. TBARS level was investigated by using millimole coefficient of absorbance (156 mmol/cm). TBARS level was expressed in nmol/ml.

### Statistical analysis

Arithmetic means and descriptive statistics of SOD and CAT activity and the concentration of GSH and level of TBARS and Fe, Cu, Mn, and Co concentration in the livers and kidneys of great tit nestlings were calculated. The results of Pearson’s chi^2^ test (Chi^2^ = 10.666, df = 28, *p* = 0.999) indicates the comparable number of samples in each groups obtained in view of existing variable grouping (environment and age of nestlings). Then we introduced the data of the three groups of nestlings from different environments (Table 1). The data did not show a normal distribution; hence, non-parametric tests were used (ANOVA Kruskal–Wallis test, followed by multiple Kruskal–Wallis test) to estimate the significance of differences in the level of oxidative stress parameters and the concentration of elements in the livers and kidneys of nestlings from different environments. The relation between SOD, CAT, GSH, and TBARS and concentrations of Fe, Cu, Mn, and Co in both organs and between organs from different environments were calculated by correlation coefficient (*R*_s_), according to the rank of Spearman test (significance level α < 0.05) (Stanisz 2006).

## Results

### Liver

We found significantly higher degrees of lipid peroxidation (1.05 ± 0.28; 1.36 ± 0.24) and CAT activity (280.95 ± 58.02; 243.72 ± 68.77) in the livers of nestlings from the Kujawy region (sodium factory and agricultural areas, respectively) than in those from the control area (TBARS, 0.759 ± 0.266; CAT, 200.12 ± 53.36). CAT activity in the liver was the highest especially in the vicinity of sodium factories. By contrast, SOD had more than 30% lower activity in the livers of nestlings from sodium factories (0.060 ± 0.027) compared to those from agricultural areas (0.090 ± 0.048). GSH concentrations in liver tissues did not differ among young tits in the studied environments (Table 2).

**Table 2:** Mean biomarker responses (superoxide dismutase [SOD] and catalase [CAT] activities and levels of total glutathione [GSH] and lipoperoxidation [TBARS] in great tit nestlings in different environments (Kujawy region: (A) sodium manufactures; (B) agricultures; (C) and control (Tuchola Forestry)

	Livers					Kidneys			
Environment	N	Mean	SD	*p*		N	Mean	SD	*p*
Fe [mg*kg^−1^]									
A	38	1523.618	677.436	**0.003**^**AB**^		38	540.547	168.070	**0.027**^**BC**^
B	44	2273.033	1070.169	**0.002**^**BC**^		44	550.553	155.961	1.000^AB^
C	41	1483.592	783.369	1.000^AC^		41	466.900	167.205	0.165^AC^
Cu [mg*kg^−1^]									
A	38	12.658	2.780	**< 0.001**^**AC**^		38	11.780	18.244	**0.001**^**AC**^
B	44	15.064	6.572	0.050^BC^		44	9.289	4.307	**0.032**^**BC**^
C	41	19.039	8.686	0.213^AB^		41	13.096	10.525	0.691^AB^
Co [mg*kg^−1^]									
A	38	0.015	0.012	**< 0.001**^**AB**^		38	0.012	0.011	**< 0.001**^**AC**^
B	44	0.027	0.016	**0.024**^**AC**^		44	0.020	0.027	**0.001**^**BC**^
C	41	0.024	0.014	0.627^BC^		41	0.031	0.022	0.202^AB^
Mn [mg*kg^−1^]									
A	38	5.359	1.296	**< 0.001**^**AC**^		38	9.676	2.100	**< 0.001**^**AC**^
B	44	5.280	1.406	**< 0.001**^**BC**^		44	10.202	2.774	**< 0.001**^**BC**^
C	41	6.836	1.318	1.000^AB^		41	16.568	6.324	1.000^AB^
SOD [U/ml]									
A	38	0.060	0.027	**0.002**^**AB**^		38	0.125	0.049	**0.003**^**BC**^
B	44	0.090	0.048	0.068^AC^		43	0.120	0.054	0.931^AB^
C	41	0.088	0.051	0.773^BC^		41	0.165	0.078	0.096^AC^
CAT [nmol/min/ml]									
A	38	280.950	58.018	**0.049**^**AB**^		38	111.643	39.841	**< 0.001**^**AC**^
B	44	243.724	68.767	**< 0.001**^**AC**^		43	141.541	49.775	**< 0.001**^**BC**^
C	41	200.123	53.356	**0.003**^**BC**^		41	65.207	33.550	0.057^AB^
GSH [μM]									
A	38	47.492	20.165	0.108^AB^		38	4.974	2.625	0.047^AC^
B	44	58.097	17.587	0.070^AC^		43	3.534	2.473	**< 0.001**^**BC**^
C	41	57.516	22.991	1.000^BC^		40	6.790	3.268	0.070^AB^
TBARS [nmol/ml]									
A	38	1.048	0.282	**< 0.001**^**AB**^		38	0.989	0.592	**0.043**^**AB**^
B	40	1.357	0.242	**0.001**^**AC**^		44	1.229	0.687	1.000^AC^
C	41	0.759	0.266	**< 0.001**^**BC**^		35	0.947	0.780	0.080^BC^

Mean values are presented as Fe, Cu, Co, and Mn concentration [mg*kg^−1^], SOD [U/ml], CAT [nmol/min/ml], GSH [μM], TBARS [nmol/ml], SD standard deviation, p − *p* value for multiple comparisons between environments (upper index): A (areas located in the vicinity of soda plants), B (agriculture sites), C (unpolluted sites). The bold p indicates a significant difference (*p* > 0.05) between environment indicated in the upper index

Fe, Cu, Co, and Mn concentration also differed in the livers from nestlings and depended on the environment (*p* < 0.05). Higher Fe concentration was found in the livers from agricultural areas (2273.03 ± 1070.17) compared to other environments (1523.62 ± 677.44 (sodium manufactures); 1483.59 ± 783.37 (control) (Table 2). However, as Table 2 shows, Cu and Mn concentration was higher in the livers of control birds as opposed to Co level (lower in sodium factory areas).

**Fig. 1:**
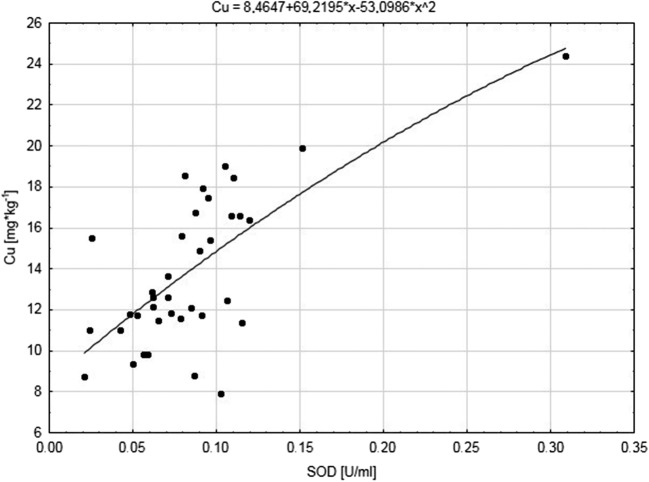
Relation between copper Cu (mg*kg^−1^) concentration and activity of superoxide dismutase SOD (U/ml) in livers of great tits from agriculture environment (B)

Significant correlations were found between the level of oxidative stress parameters and concentrations of selected transition metals (*p* < 0.05) (Table 3). Increased SOD activity seemed to depend mostly on Mn concentration in all examined environments. Furthermore, SOD activity showed positive relations with Co (sodium factories, agricultural areas) and Cu (agricultural areas) (Fig. 1). We stated both positive (sodium factories, agricultural areas) and negative (control) correlations between CAT activity and Mn level. CAT activity was also positive in relation to Cu and negative in relation to Co in birds from sodium manufacturing areas. We found positive correlations with Co (sodium factories, control), Fe (agricultural areas), and Mn and negative with Fe (sodium factories), Mn (agricultural areas), and Cu (agricultural areas, control) for GSH concentrations. Lipid peroxidation was positively correlated with Fe (sodium factories), and negatively with Co (agricultural areas, control) and Mn (control) (Table 3).

**Table 3:** Spearman coefficient analysis of biomarker responses: superoxide dismutase (SOD) and catalase (CAT) activities and levels of total glutathione (GSH) and lipoperoxidation (TBARS) on cooper (Cu), cobalt (Co), iron (Fe), and manganese (Mn) concentrations in the livers and kidneys of great tit nestlings from different sites

Livers				Kidneys			
Relations	*N*	*R*_s_	*p*	Relations	*N*	*R*_s_	*p*
*Sodium manufactures (A)*							
SOD and Mn	38	0.40	0.013	SOD and Co	38	0.38	0.019
CAT and Mn	38	0.42	0.009	CAT and Mn	38	0.34	0.038
CAT and Co	38	− 0.38	0.020	GSH and Fe	38	0.32	0.050
CAT and Cu	38	0.40	0.014	GSH and Co	38	− 0.60	< 0.001
GSH and Fe	38	− 0.34	0.039	GSH and Cu	38	− 0.44	0.006
GSH and Co	38	0.68	< 0.001	MDA and Fe	38	0.69	< 0.001
MDA and Mn	38	0.40	0.014	MDA and Co	38	− 0.83	< 0.001
MDA and Fe	38	0.49	0.002	MDA and Cu	38	− 0.43	0.007
MDA and Co	38	− 0.32	0.049				
*Agricultures (B)*							
SOD and Mn	44	0.37	0.013	SOD and Fe	43	− 0.35	0.021
SOD and Co	44	0.46	0.002	SOD and Co	43	0.49	0.001
SOD and Cu	44	0.52	0.000	SOD and Cu	43	0.48	0.001
CAT and Mn	44	0.44	0.003	CAT and Co	43	− 0.58	< 0.001
GSH and Mn	44	− 0.43	0.004	CAT and Cu	43	− 0.68	< 0.001
GSH and Fe	44	0.37	0.015	GSH and Co	43	− 0.72	< 0.001
GSH and Cu	44	− 0.50	0.001	GSH and Cu	43	− 0.58	< 0.001
				MDA and Co	44	− 0.67	< 0.001
				MDA and Cu	44	− 0.66	< 0.001
*control environment (C)*							
SOD and Mn	41	0.34	0.031	SOD and Mn	41	0.51	0.001
SOD and Co	41	0.35	0.025	SOD and Fe	41	− 0.49	0.001
CAT and Mn	41	− 0.50	0.001	SOD and Cu	41	− 0.39	0.011
GSH and Co	41	0.39	0.011	CAT and Fe	41	0.43	0.005
GSH and Cu	41	− 0.33	0.033	CAT and Cu	41	0.43	0.005
MDA and Mn	41	− 0.44	0.004	MDA and Mn	35	− 0.79	< 0.001
MDA and Co	41	− 0.57	< 0.001				

*r*—Spearman’s correlation coefficient; *p*—probability level

### Kidneys

We determined higher Fe concentrations in the kidneys of young tits from agricultural areas (550.55 ± 155.96) than in those from control (466.9 ± 167.205). However, the level of Cu, Co, and Mn was higher in birds from the control environment as compared to both Kujawy areas studied (*p* < 0.032; *p* < 0.000). SOD activity and GSH concentrations were higher in birds from the control environment (*p* < 0.05). By contrast, CAT activity and TBARS concentration were higher (*p* < 0.000; *p* < 0.043) in agricultural areas than near sodium factories and controls (CAT) and sodium factories (TBARS) (Table 2).

**Fig. 2:**
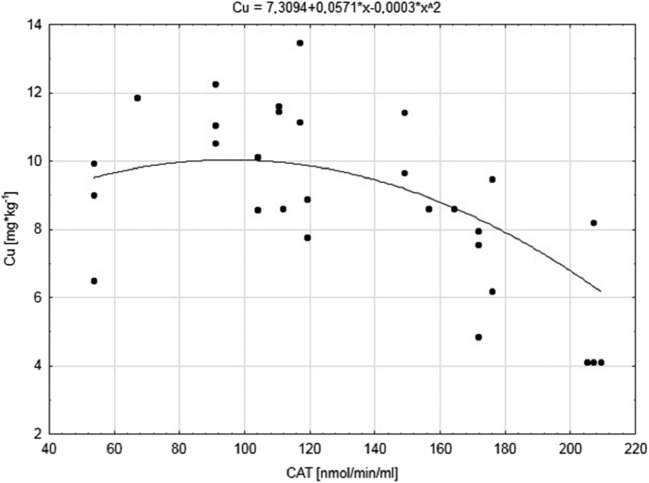
Relation between copper Cu (mg*kg^−1^) concentration and activity of catalase CAT (nmol/min/ml) in kidneys of great tits from agriculture environment (B)

We found especially significant relations (*p* ≤ 0.021) between oxidative stress parameters (positive: SOD; negative: CAT, GSH, TBARS) and Co and Cu concentrations in the kidneys from nestlings from the agriculture area (Table 3, Fig. 2). Similarly, Co concentration was also positively correlated with SOD, and negatively with CAT and TBARS in birds from near sodium factories. Increased GSH and TBARS concentrations in sodium factories and SOD and CAT activity in control seemed to depend on increased Cu level. We found positive relations between Fe level and GSH and TBARS concentrations near sodium factories and negative relations with SOD activity in birds from agricultural areas. Furthermore, CAT and SOD activity were stimulated and blocked, respectively, by Fe level in birds from control. For Mn level, only relations with CAT were stated (*R*_s_ = 0.34; *p* ≤ 0.038) in birds from near sodium factories, and with SOD (*R*_s_ = 0.51; *p* ≤ 0.001) and TBARS (*R*_s_ = − 0.71; *p* ≤ 0.000) in the control group (Table 3).

### Element concentrations and level of biochemical indicators of oxidative stress: relations between organs

Liver and kidneys TBARS were found to significantly positively correlate with one another (within all environments, particularly in sodium manufacturing areas [*R*_s_ = 0.64; *p* < 0.001]). CAT activity in the livers positively correlated with CAT activity in the kidneys for chicks from the agricultural area (*R*s = 0.54; *p* < 0.001). Furthermore, GSH level showed a negative relation between the chicks’ organs from near sodium factories. We found positive correlations in concentrations of Mn (all environments), Fe (sodium manufacture, control), and Co (sodium manufactures, agricultures) between organs. Concentration of Cu was only negatively correlated between the livers and kidneys of chicks from near sodium factories (Table 4).

**Table 4:** Spearman coefficient analysis of superoxide dismutase (SOD) and catalase (CAT) activities and levels of total glutathione (GSH) and lipoperoxidation (TBARS), cooper (Cu), cobalt (Co), iron (Fe), and manganese (Mn) concentrations between liver and kidneys of great tit nestlings

Livers and kidneys			
Relations	*N*	*R*_s_	*p*
*sodium manufactures (A)*			
SOD and SOD	38	0.026	0.877
CAT and CAT	38	0.161	0.335
GSH and GSH	38	− 0.420	**0,009**
MDA and MDA	38	0.639	**< 0.001**
Mn and Mn	38	0.601	**< 0.001**
Fe and Fe	38	0.502	**0.001**
Co and Co	38	0.762	**< 0.001**
Cu and Cu	38	− 0.367	**0.023**
*agricultures (B)*			
SOD and SOD	43	0.222	0.153
CAT and CAT	43	0.538	**< 0.001**
GSH and GSH	43	− 0.123	0.433
MDA and MDA	40	0.380	**0.016**
Mn and Mn	44	0.56	**< 0.001**
Fe and Fe	44	0.27	0.081
Co and Co	44	0.44	**0.003**
Cu and Cu	44	0.01	0.931
*control environment (C)*			
SOD and SOD	41	0.25	0.109
CAT and CAT	41	0.24	0.138
GSH and GSH	40	0.76	**< 0.001**
MDA and MDA	35	0.44	**0.008**
Mn and Mn	31	0.48	**0.002**
Fe and Fe	41	0.54	**< 0.001**
Co and Co	41	0.21	0.193
Cu and Cu	41	0.20	0.201

*r*, Spearman’s correlation coefficient; *p* probability level

The bold relations are statistically significant (*p* < 0.05)

## Discussion

Changes in concentration of chemical elements in the nestlings of great tits depend on their level in the food chain and individual predispositions. It is quite significant that invertebrates, which are one of the major sources of the birds’ food, are particularly exposed to heavy metals (Carpene et al. 2006; Roodbergen et al. 2008), and thus, birds are very sensitive to changes occurring in the structure of the environment (Savard et al. 2000). In this work, invertebrates (including insects) from agrocenosis and areas located near sodium plants probably accumulate pesticides and heavy metals getting into the environment as a result of agricultural practices and the sodium industry (Kamiński et al. 2016). Interestingly, our results (this paper) indicated significantly higher concentrations of Fe in both studied organs of chicks colonising agricultural areas, while Cu and Mn were higher in the control environment, and Co was lowest in the environment adjacent to the sodium plants (Table 2).

It is also difficult to indicate the toxic level of trace elements for insectivorous birds living in natural conditions. However, concentrations of the examined metals (this paper) were similar, lower or even higher than other results of research on wild birds by Llacuna et al. (1995). This shows especially higher average Cu and Fe concentrations in the liver and kidneys of adult great tits in Spain in relation to the results obtained in this paper (Table 2). In turn, we can conclude that the great tit’s chicks (this paper) accumulated higher concentrations, especially of Fe and Mn in their tissues, than rook (*Corvus frugilegus*) nestlings (1–13 days old) from agricultural and rural areas close to the Siedlce region (south-central Poland) (Orłowski et al. 2012). On the other hand, the research of Deng et al. (2007) carried out on adult great tits in settling areas of Badachu Park (Beijing, China) confirmed approximate concentrations or lower Mn and Cu levels as compared to the results obtained in this paper (Table 2), especially Cu in the kidneys and Mn in the livers. In turn, Mn concentration in the kidneys and Cu in the livers were higher in Great Tits examined by Deng et al. (2007). Differences in the concentration of elements in individual bird organs may be related to: the ability of the bird species to bioaccumulate elements, the age of the individual, and the degree of environmental pollution. The analytical method used to assess metal concentration is also significant.

Many reports indicated the contribution of trace metals in redox reactions, and thus the formation of ROS (Koivula and Eeva 2010; Jomova et al. 2012; Rainio et al. 2013; Kharroubi et al. 2014). The consequence of these processes is the damage of all classes of molecular components of cells, i.e. lipids, DNA, and proteins (Kamiński et al. 2009; Rainio et al. 2015; Sánchez-Virosta et al. 2019). The result of the excess of ROS in cells (oxidative stress) is the modulation of the activity of antioxidant system to restore homeostasis (Halliwell and Gutteridge 2015; Sánchez-Virosta et al. 2019). According to our prediction, our data (this paper) suggest increased generation of ROS, and consequently higher level of damage of lipids in the organs of nestlings studied in the polluted Kujawy region as opposed to those from Tuchola (Table 2). We found higher SOD activity as opposed to CAT in both examined organs of nestlings in the control environment relative to environments located in the Kujawy region (Table 2). Such results may suggest the possibility of discriminating SOD function in polluted environments in consequence of increased level of ROS or activation of CAT, which decomposes H_2_O_2_ very efficiently, and thus, SOD is not activated. Similarly, Berglund et al. (2007) also showed a coordinated relationship between these two enzymes in the livers of pied flycatcher (*Fiecedula hypoleuca*) from a polluted environment close to sulphide or smelting industry in northern Sweden. They confirm that the efficient operation of CAT (H_2_O_2_ decomposition) enabled the SOD to function at an appropriate level (Berglund et al. 2007). Secondly, as with SOD activity, the level of GSH in the kidneys of great tit nestlings was also higher in the control area as compared to the anthropically changed Kujawy region (this paper). This argument, as well as the high level of TBARS in organs of chicks colonising the Kujawy region, might tend confirm the impairment of the defence mechanisms of the chicks from polluted areas. However, differences in GSH concentrations in the livers of birds from various environment were not found (Table 2), just as in the work of Isaksson et al. (2005), Berglund et al. (2007), Isaksson et al. (2009), Koivula et al. (2011), and Rainio et al. (2013). Furthermore, Isaksson et al. (2005) did not find differences in the ratio between oxidised and reduced glutathione (GSSG:GSH) concentration as an indicator of stress in blood plasma of 13-day-old great tit nestlings depending on the different environment (urban, suburban, rural) in south-western Sweden, near Gothenburg. They indicated generally higher GSH levels in adult *P. major* than in nestlings, and the higher rate of GSSG:GSH in blood plasma of adults from urban areas was greater than in adults from rural ones. Probably, as noted by Isaksson et al. (2005), chicks do not have a sufficiently developed defence system and thus fail to adapt to various stressors, e.g. limitation of cysteine (amino acid necessary to GSH synthesis). Similarly to their conclusions, we can confirm that GSH could constitute a long-term up-regulation indicator of this antioxidant reservoir in the livers of chicks. However, to fully interpret our results, it is necessary to further examine the GSSG:GSH ratio, which was impossible due to the small amount of material tested. To sum up, enzymatic defence mechanisms in the livers and kidneys of chicks of *P. major* are shaped in response to changes in various environmental factors, i.e. concentration of trace metals. Thus, the reduction of ROS determines the effective operation of complementary antioxidant mechanisms as shown by Sánchez-Virosta et al. 2019. The confirmation of the mutual cooperation of individual components of the antioxidant system may be their interactions with transition elements (Fe, Cu, Mn, Co) both in the livers and kidneys and between organs of chicks from different habitats (Table 2, Table 3, Table 4, Figs. 1 and 2). The type and direction of these relations (Tables 3 and 4) particularly depends on habitat conditions but also the concentration of elements and their interaction. Especially strong relationships (*R*_s_ > 0.5) were observed in the concentration of Co with GSH in both organs from sodium manufacturing areas; Cu with SOD, GSH in the livers, and Co and Cu with CAT, GSH, and TBARS in the kidneys of chicks from agricultural areas (Table 3, Figs. 1 and 2). In turn, in the reference environment, Mn and Co affect the activity of CAT and TBARS, respectively, in the livers, while in the kidneys, Mn was correlated with SOD and TBARS (Table 3). The above relationships confirm the clear contribution of individual transition elements of activation of the antioxidants depending on the nestlings habitat (Co [A]; Co, Cu [B]; Co, Mn [C]). The destabilisation of mineral management, high pH, and salinity within ecosystems in the Kujawy region determines the reduced bioavailability of trace elements, i.e. Cu and Mn, necessary for the growth and proper condition of the chicks, and thus the development of an efficient default system. The examined transition metals are involved in the activation of SOD, CAT, or GSH as evidenced by numerous interactions (Table 3). Interestingly, the same element can have a completely different effect on the components of the antioxidant system in chicks from the same environment. For example, in nestlings colonizing agrocenosis, Cu was found to correlate positively with SOD and negatively with GSH, while in the kidneys, it correlated positively with SOD but negatively with other parameters (Table 3, Figs. 1 and 2). Within each of the three research areas, only the concentration of Mn in the livers significantly correlated with its concentration in the kidneys, as did individual indicators of oxidative stress. The effects of ROS are visible in both the livers and kidneys of the chicks in each environment (Table 4).

Hence, measuring the level of a single biomarker of oxidative stress cannot be a determinant of the way the entire antioxidant system functions, which was also noted earlier in works by Berglund et al. (2007), Koivula and Eeva (2010), Espín et al. (2014), and Halliwell and Gutteridge (2015). In the natural environment, birds are exposed to many, sometimes, immeasurable factors that interact with each other, significantly affecting changes in the ecophysiological response that are often difficult to interpret. Therefore, although the literature data do not clearly indicate that the levels of the elements examined in both the livers and kidneys (Table 2) are toxic (Kabata-Pendia 2010), it can be assumed that the activation of the antioxidant system in the chicks depends on the environment as a result of many factors (not only transition metals), which assumption is also unexplored in this work. Additionally, we can suspect that the concentration of Fe increases the lipoperoxidation level in both organs from chicks located near the sodium plants. On the one hand, Fe stimulated the growth of GSH in the liver, but it also decreased its growth in the kidneys (Table 3). In other environments, the relationship between the individual elements (Cu, Co, Mn) and the level of lipoperoxidation has instead been negative (Table 3). These results indicate a special contribution of iron. The generation of ROS confirms our earlier observations of the existence of various antioxidant mechanisms in both organs and may be dictated by the difference in the concentration of this element. However, research carried out on pied flycatchers in Sweden confirmed an increase in the level of ROS and also an increase in enzymes (CAT, GR) in the livers of nestlings from the polluted habitat. The concentration of Fe is the cause of oxidative stress in chicks of the pied flycatcher and changes in the operation of ROS deactivating mechanisms (Berglund et al. 2007). Similarly, the role of transition elements in generating ROS is emphasised by Kamiński et al. (2009). They confirmed the level of CAT, GPx, SOD, and GR activities and TBARS concentration in blood of nestlings (19–54 days) of white stork (*Ciconia ciconia*), especially from a polluted region near Głogów, where the industry is connected with output and production, i.e. copper. The concentrations (mg/kg) of Mg (6000), K (3.8), Cu (10.9), Mn (47.6), Co (5.6), Zn (9.7), but particularly of toxic metals Pb (7.2), and Cd (2.2) in the blood of chicks may affect their antioxidant response and suggest that they do not have suitable conditions for proper growth and development (Kamiński et al. 2009). Conversely, research by Koivula et al. (2011) suggests that metals do not directly increase oxidative stress in nestlings of tits (Harjavalta, Finland), where Cu, Ni, As, Zn, Pb, and sulphur oxides were a source of environmental contamination. However, changes caused in the diet of birds in these areas as result of environmental pollution indirectly increase nestling mortality. Apart from the concentration of the above metals, the quantity of accessible carotenoids in the population of invertebrates can decrease, which probably indirectly contributes to increased oxidative stress in birds (Eeva et al. 2005, 2008). Similarly, research by Isaksson et al. (2009) on adult great tits (the western sea coast of Sweden, Gothenburg) evidenced the lack of essential differences in CAT activity in the livers and TBARS concentration in the lungs among birds from urban (Gothenburg) and rural areas (40–50 km south of Gothenburg). However, they found higher content of carotenoids in the livers of urban males rather than in rural males, so the concentration of these antioxidants is not only habitat but also sex-dependent (Isaksson et al. 2009). Comparing our results (present study) with others (Berglund et al. 2007; Kamiński et al. 2009; Rainio et al. 2013; Herrera-Dueñas et al. 2017), it appears that antioxidant defence responds differently depending on environmental pollution and bird species but also on individual predisposition to the activity of antioxidant systems.

It can be concluded that changes in the metabolism of chemical elements in great tit nestlings are due to environmental stress and cause significant eco-physiological responses in bird organisms connected with changes in the activity of the antioxidant system. On the other hand, these changes are species-dependent and conditioned by the degree of environmental pollution, food availability, and study material, which makes interpretations considerably difficult. We can suggest that young great tits in polluted areas face more difficult conditions for growth than the populations living in areas near Tuchola. Probably, the disturbed management of essential elements in Kujawy prevents effective protection against oxidative stress and may manifest as a high level of lipoperoxidation.

The heavily anthropically affected environment forces the development of appropriate reactions to preserve a balanced mineral economy and activate an antioxidant system that protects against changes resulting from oxidative stress, and which may occur in every cell. These mechanisms determine survival, and on the other hand, affect the condition of the chicks and the reproduction of these birds (Costantini et al. 2015; Isaksson 2015).

## Conclusions

great tit nestlings do not have optimal conditions for growth and development in polluted environments. We found a high degree of lipid peroxidation, as well as lower activity of SOD in both the livers and kidneys, and a lower level of GSH in kidneys of great tits located in Kujawy relative to those from a reference environment. This may suggests dysfunction of the antioxidant system and increased exposure of chicks to the effects of oxidative stress in the Kujawy region. On the other hand, CAT activity was higher in the livers and kidneys of chicks from Kujawy, and the level of GSH did not differ between environments. Therefore, we need more information to confirm our prediction about the functioning of the antioxidant system. Fe concentrations could particularly influence peroxidation of lipids in polluted areas. The level of oxidative stress biomarkers (SOD, CAT, GSH) can be determined by the level of transition elements such as Fe, Cu, Mn, or Co, but probably indirectly by other factors (interaction with toxic metals, pesticides, fertilisers). Mutual regulation of the antioxidant system in connection with the action of heavy metals still remains an unexplored subject requiring more detailed research.
